# Considerations for protein and amino acids in standardized reference diet for parthenogenetic marbled crayfish *Procambarus virginalis* model organism

**DOI:** 10.1038/s41598-024-58304-3

**Published:** 2024-07-16

**Authors:** Koushik Das, Koushik Roy, Jan Mráz, Miloš Buřič, Antonín Kouba

**Affiliations:** Faculty of Fisheries and Protection of Waters, South Bohemian Research Center of Aquaculture and Biodiversity of Hydrocenoses, University of South Bohemia in České Budějovice, Zátiší 728/II, 389 25 Vodňany, Czech Republic

**Keywords:** Laboratory model, Laboratory diet, Ideal protein, Amino acids, Nutritional control, Lipids, Proteins

## Abstract

The concept of a standardized reference diet (SRD) is used in laboratory model organisms to ensure nutritional control between studies and laboratories. Although models using the genetically identical, all female parthenogenetic marbled crayfish (*Procambarus virginalis*) are growing in popularity, research into nutrition in this species still has many knowledge gaps. To fast track the development of a SRD in terms of protein and amino acids (SRD_protein_) for this species, we first analyzed the composition of its body amino acids to determine the ideal protein concept (IPC) of indispensable amino acids in wild-caught *P. virginalis* (which had an unusually high preponderance of leucine and arginine). Then, we strategically evaluated three common clusters of types of fish feed: (1) ornamental fish feed (SER) fortified with a naturally occurring alga (*Spirulina*). This type of feed was protein-high in arginine and leucine (SER + SPI) that fulfils the species’ IPC for iso-protein (~ 40%), iso-phosphorus (~ 0.8%) and near iso-energetic (~ 475 kcal 100 g^−1^); (2) freeze-dried live feed consisting of chironomid larvae (CHI) fortified with *Spirulina* (CHI + SPI) that fulfils the IPC for iso-protein (~ 46%), iso-phosphorus (~ 0.7%) and near iso-energetic (~ 405 kcal 100 g^−1^); and (3) a commercially standardized ‘starter diet’ for carnivorous fish larvae (FISH) and post-larval shrimps (SHRIMP) with iso-protein (~ 56%) and iso-phosphorus (~ 1.6%). A total of six diets, embracing a diverse range of proteinaceous feeds, were used in a 100-day ad libitum feeding and growth trial. The FISH group outperformed all the other groups (*p* < *0.05*) and our exploratory multivariate analysis revealed an ideal demand of > 44% protein (tailored to deliver high arginine 3% and leucine 4%, followed by the usual lysine > 3.5% and methionine 1.2%) but also the lowest carbohydrate level (21%). For SRD_protein_, our findings show that the FISH diet is ideal and suggest the possibilities of using a CHI + SPI diet for further optimization (more economic use of protein and phosphorus).

## Introduction

Model organisms are extensively studied non-human species that can be used to understand a range of biological phenomena. They can help develop data and hypotheses with broad implications extending beyond their specific domain of study, which can encompass other organisms, including humans. A number of commonly recognized model species are used for biomedical research to achieve consistent results, which implies that culture conditions and husbandry must be standardized^1^. In laboratory animals, diet is a significant component that influences disease resistance, growth, reproduction and how animals respond to manipulation during experiments^2,3^.

Habitually, dietary choice in laboratories relies on anecdotal evidence and uses anonymous diets or diets designed for other species that often lack comprehensive compositional analysis. Consequently, the degree of experimental nutritional control is limited^3^. Several diets for aquatic organisms are commercially available, most of which are based on general fish nutritional guidelines. However, the development of standardized or reference diets remains a challenge in laboratory animal husbandry, particularly due to the underestimation of the importance of these standards and lack of knowledge of nutritional requirements^4,5^.

There is often a lack of knowledge regarding the nutritional requirements of newly introduced model organisms^6^. The composition of amino acids^7^ and fatty acids (Part 2 of this study) can substantially influence reproductive and somatic performance^6,8–10^. For example, in zebrafish *Danio rerio* and Japanese medaka *Oryzias latipes*, an SRD has been established after decades of research and now can be used to mitigate inter-laboratory and inter-experiment differences^3,11^. Despite this, for killifish *Nothobranchius furzeri*^12^ and marbled crayfish *Procambarus virginalis* (present study) such efforts are currently still at an preliminary stage.

The marbled crayfish is a recently evolved parthenogenetic freshwater crayfish species ^13^ that has invaded diverse habitats in Europe, Asia, Madagascar and North America^14^. It is a triploid and obligate parthenogenetic crustacean, first detected in the pet trade in Germany (~ 25 years ago), that is derived from the sexually reproducing diploid slough crayfish *Procambarus fallax*^15,16^. The marbled crayfish has been identified as an invasive species of EU concern since it threatens native crayfish populations due to its prolific, all-female monoclonal population-forming capability and wide tolerance to environmental stress^14,17,18^. In spite of its growing importance as a model organism in biomedical research^19,20^, to date no nutritional control is used in experiments with this model organism. However, previous research into procambarid crayfish nutrition^21,22^ laying the foundation for the present study has recently emerged.

Traditionally, laboratories use frozen or live chironomid larvae with or without grated carrots as the basis for crayfish diets. Ornamental/aquarium fish feed (flakes) such as Sera Granugreen and Tetra Wafer Mix are also used^17,22–25^. However, the nutritional value of these feeds—including chironomids—is known to be insufficient for marbled crayfish^22^. We postulated that laboratory model marbled crayfish would depend less on live food (as discussed^12^) and less on ornamental fish feed due to less standardarized or less strict formulation criteria compared to aquafeed for commercial (aquaculturally valuable) aquatic animals. The aims of this study were (a) to understand the dietary preferences of marbled crayfish and dietary protein needs based on whole body protein profiles of wild-caught animals; (b) to compare growth, body size (or maturity) and survivability responses under traditional diets (with a basic amino acid profile) versus meticulously formulated chosen diets (with an advanced amino acid profile); (c) to identify and propose an SRD protein for marbled crayfish that could be further validated with a semi-purified research diet. The managerial implications for this study include the fast-tracking of SRD development for the marbled crayfish model and the stimulation of research into its nutritional needs.

## Results

### Macronutrients and energy influence on somatic growth

**Figure 1:**
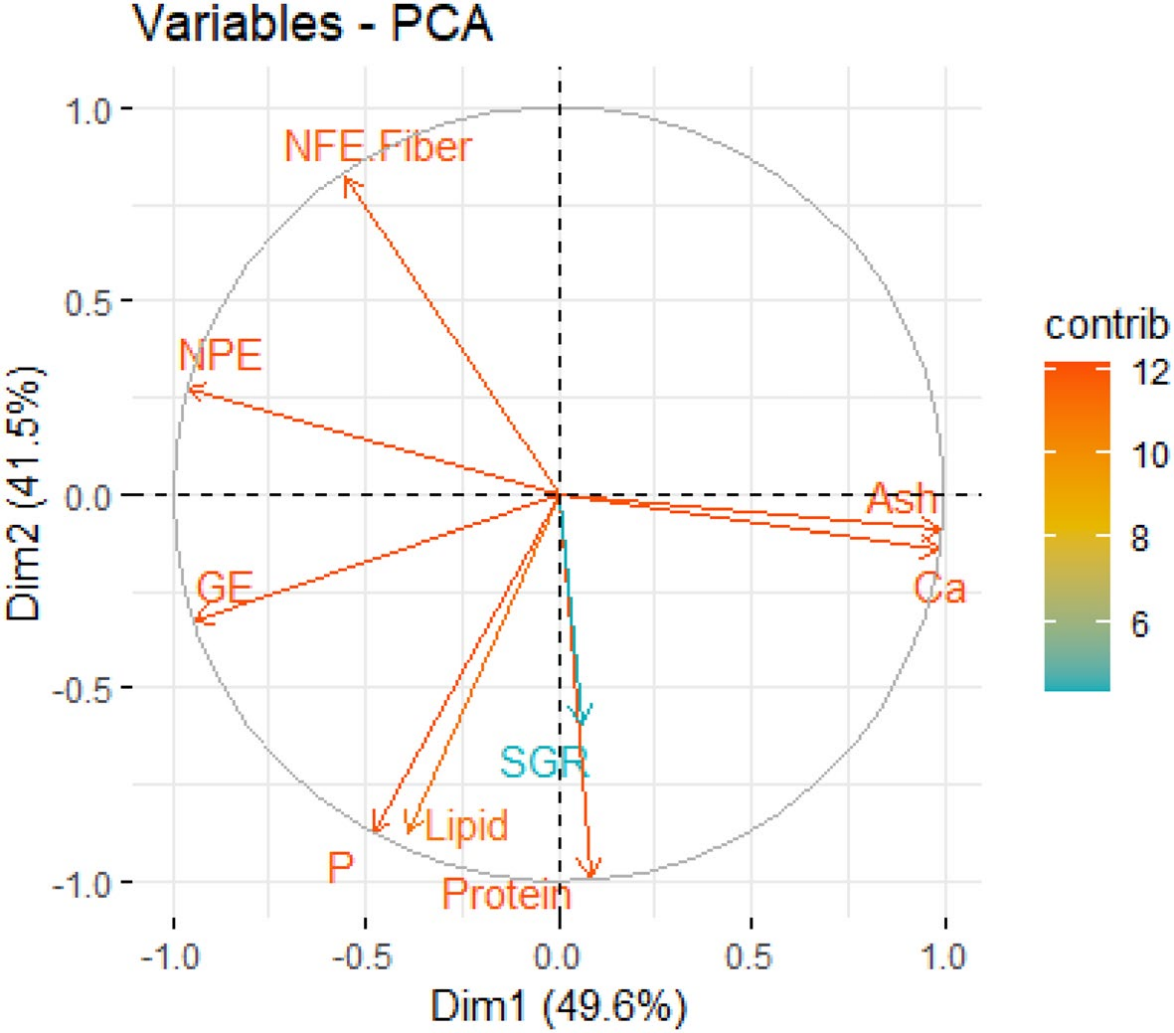
Variables of the principal component analysis (PCA) biplot showing specific growth rate (SGR) vectors concerning nutrient and energy parameters of the diets (listed in Table 1). Anti-clockwise: NFE. Fibre = carbohydrates, NPE = non-protein energy, GE = gross energy, P = phosphorus, Ca = calcium. X, Y axis: Dim = Dimension, Legend: contrib = contribution.

The replicate-wise results of the specific growth rate (SGR) of 0.03 g initial body-weight marbled crayfish after 100 days of ad libitum feeding at 21 ± 1 °C under different experimental diets are given in Table 1. The FISH diet had significantly higher SGR (Tukey HSD *p* < *0.05*) than either the traditional laboratory SER or the CHI diets, as derived from the different protein contents. The rest of the diets were comparable to each other. Interestingly, at the highest protein levels, between the iso-protein diets FISH and SHRIMP, the FISH diet had better SGR (Tukey HSD *p* < *0.05*). Interpretation using a principal component analysis (Fig. 1) in dimension-1 explains almost 50% of the variability in the overall dataset, which suggests that there is a positive multicollinearity between SGR and protein, phosphorus and the lipid content of our tested dietary range. By contrast, carbohydrate (NFE) was most negatively related to growth (SGR). The non-protein energy (NPE vector) was most closely driven by carbohydrates (NFE vector) rather than lipids; the NPE was weakly negatively correlated to SGR (Fig. 1). Overall, the patterns suggest a ketogenic diet profile—defined as a high protein intake (including phosphorus) and calories derived from mostly protein and lipids but not carbohydrates—is preferable for marbled crayfish SRD development.

**Table 1:** Macronutrient and energetic composition of the experimental diets, including their growth response

Cluster*	Diet	Replicate	Protein (g 100 g^−1^)	Lipid (g 100 g^−1^)	Ash (g 100 g^−1^)	NFE (g 100 g^−1^)	GE (kcal 100 g^−1^)	NPE (kcal 100 g^−1^)	GE: protein (kcal g^−1^)	Ca (g 100 g^−1^)	P (g 100 g^−1^)	SGR (% day^−1^)
I	SER	1	38.8	5.3	4.9	51.0	478.5	259.6	12.3	0.7	0.8	3.21
	SER	2										2.77
	SER	3										2.60
	SER + SPI	1	41.4	3.9	5.7	49.0	471.7	238.2	11.4	0.9	0.8	3.74
	SER + SPI	2										3.60
	SER + SPI	3										3.15
II	CHI	1	46.4	5.0	26.9	21.7	398.1	136.4	8.6	4.1	0.6	3.49
	CHI	2										3.25
	CHI	3										2.79
	CHI + SPI	1	46.9	3.3	21.3	28.5	412.8	148.3	8.8	3.4	0.7	3.89
	CHI + SPI	2										3.23
	CHI + SPI	3										3.45
III	FISH	1	56.0	10.9	11.9	21.2	505.9	190.0	9.0	1.9	1.6	4.22
	FISH	2										4.08
	FISH	3										3.70
	SHRIMP	1	55.5	13.5	9.1	21.9	530.5	217.4	9.6	1.6	1.6	3.54
	SHRIMP	2										3.46
	SHRIMP	3										3.12

***Diet description:*** SER, Sera Granugreen; SER + SPI, Sera Granugreen + Spirulina; CHI,  Chironomids; CHI + SPI,  Chironomids + Spirulina; FISH, Fish feed; SHRIMP, Shrimp feed. Abbreviation: NFE, carbohydrate excluding fibre (< 1% in all diets); GE, gross energy; NPE, non-protein energy; Ca, calcium; P,  phosphorus; SGR, specific growth rate. All values are expressed on a 100% dry-matter basis. All feed had consistent dry-matter values (91–93%).

*Cluster 1 ornamental fish feed (unfortified and fortified for ideal protein concept): similar ~ 40% protein, ~ 0.8% phosphorus, ~ 475 kcal gross energy 100 g^−1^. *Cluster 2* freeze-dried live feed (unfortified and fortified): similar ~ 46% protein, 0.7% phosphorus, ~ 405 kcal gross energy 100 g^−1^. *Cluster 3* commercial ‘starter’ feed (carnivorous fish larvae and shrimp post-larvae): similar ~ 56% protein, 1.6% phosphorus.

**Figure 2:**
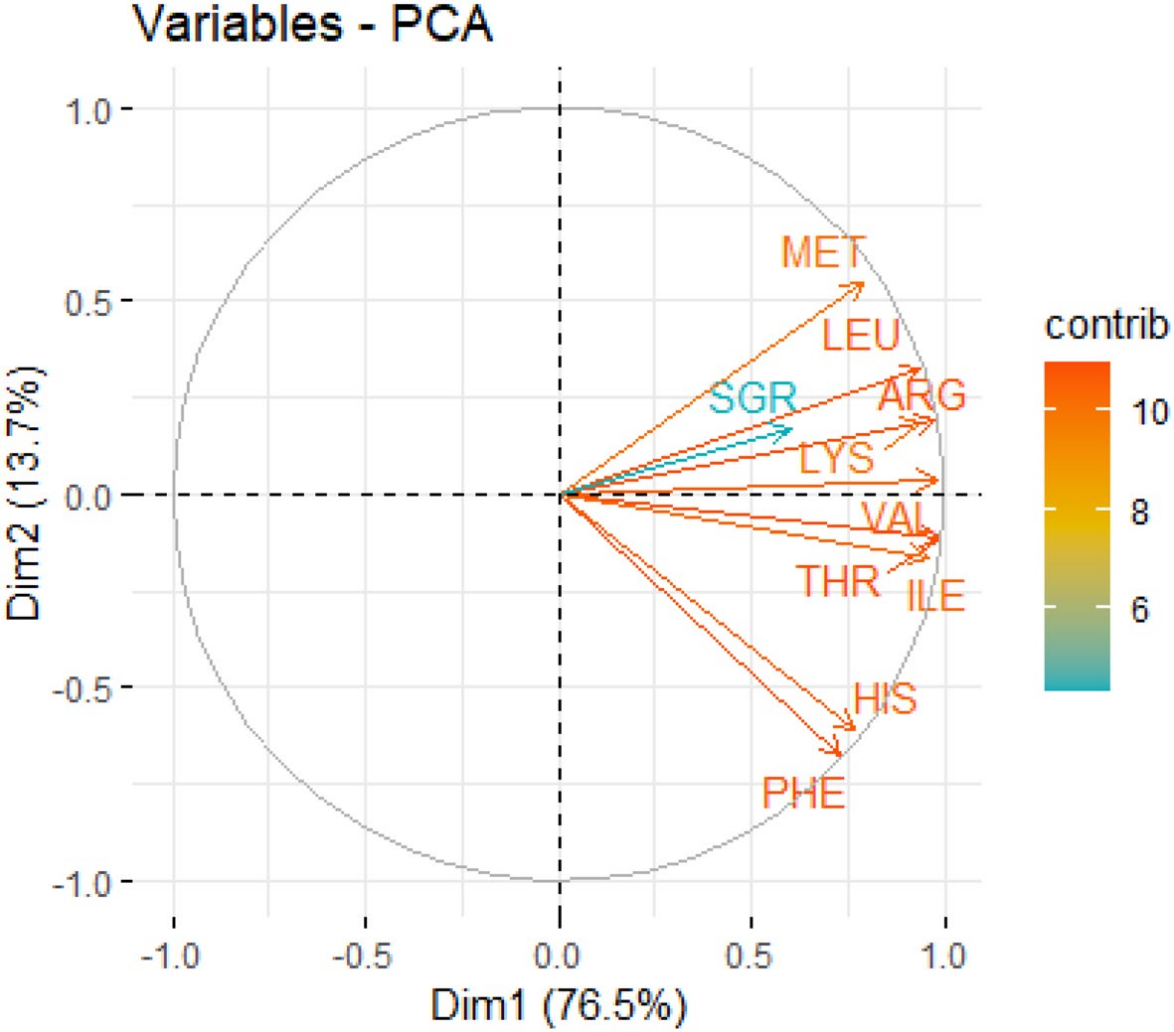
Variables of the principal component analysis (PCA) biplot showing specific growth rate (SGR) vectors concerning indispensable amino acids (IAAs) supplied from the diets (listed in Table 2). From top to bottom: methionine (MET), leucine (LEU), arginine (ARG), lysine (LYS), valine (VAL), threonine (THR), isoleucine (ILE), histidine (HIS), and phenylalanine (PHE). X, Y axis: Dim = Dimension, Legend: contrib = contribution.

**Figure 3:**
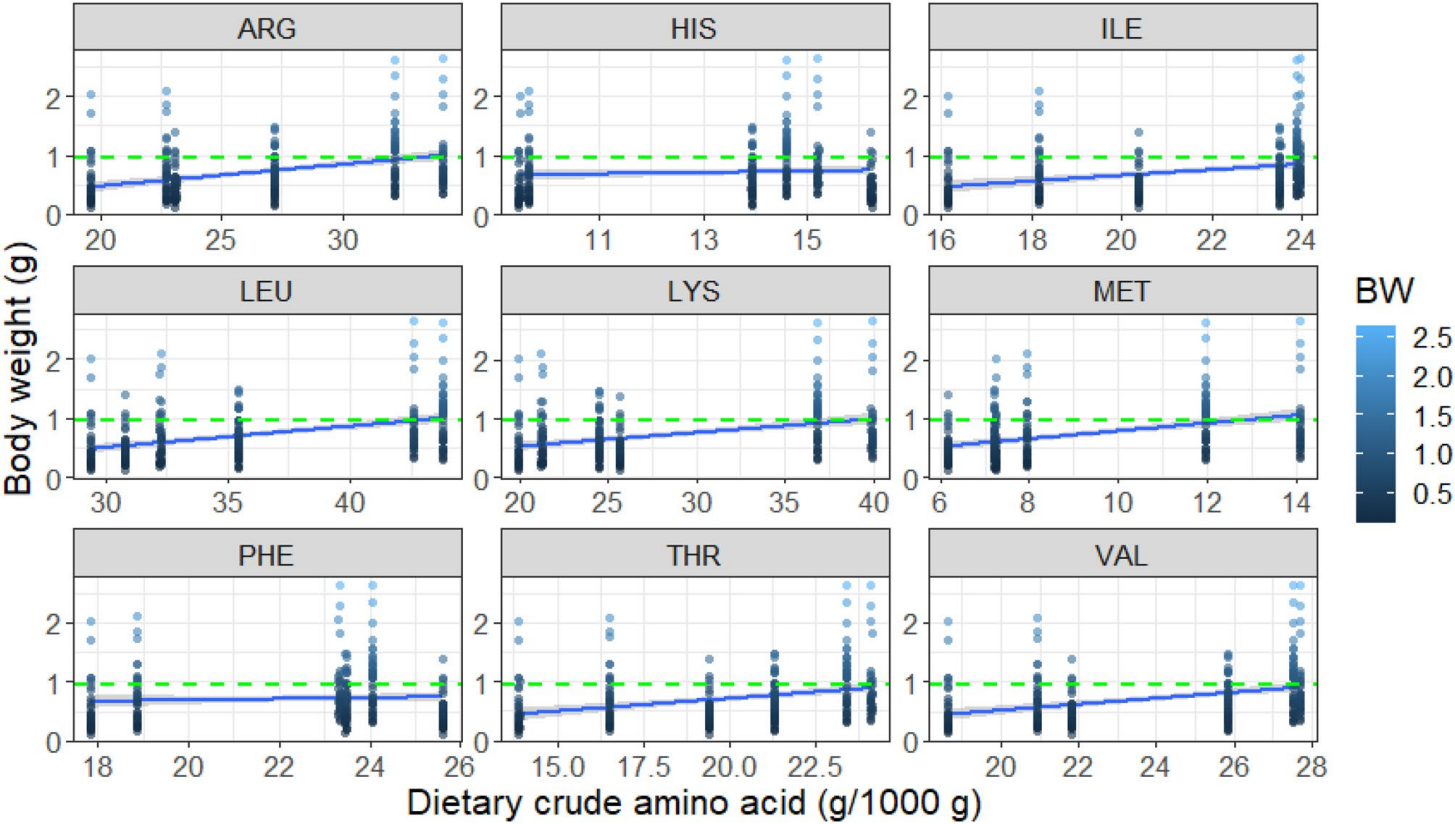
Dose–response of indispensable amino acids (IAA) to final body weight (BW) in marbled crayfish (starting body weight 0.03 g). All amino acids except histidine and phenylalanine were *p* < *0.01*. McFadden’s R^2^ value of individual models (from left to right)—Arg: 0.15; His: not generated; Ile: 0.09; Leu 0.18; Lys 0.13; Met 0.16; Phe: not generated; Thr 0.10; Val: 0.13. The green horizontal line is the benchmark for the top 25% of individuals in terms of body weight (≥ 0.96 g), from 0.03 g initial weight after 100 days at 21 °C.

Amongst the proteins, only the indispensable amino acids (IAAs) cannot be synthesized de novo by crayfish and need to be supplied in food (unlike the dispensable amino acids). Our exploratory PCA shows all IAAs were positively clustered around SGR (Fig. 2). The strongest multi-collinearity (almost overlapping) with the SGR vector were the arginine, lysine and leucine vectors. A summary of bi-variate GLM between individual IAA (independent variable) and final BW (dependent variable) is given in the Supplementary ﻿information Table S7. At a strict alpha-level (*p* < 0.01), all IAAs except histidine and phenylalanine contributed very significantly to the final body weight. Summing up McFadden’s R^2^ (see caption, Fig. 3), leucine and arginine together explained 33% of the variance in body weight. Furthermore, up to 50% of the BW variance could be explained when lysine and methionine were also considered. Leucine, arginine, lysine and methionine are thus proposed as the most critical IAAs for marbled crayfish SRD_protein_. A dose–response model between dietary IAAs and final body weight is given in Fig. 3. Thresholds of ~ 30 g arginine, ~ 40 g leucine, ~ 35 g lysine and ~ 12 g methionine per 1000 g feed could achieve more frequently (with ≥ 50% probability) body weights in the top 25% of all individuals (> 0.96 g; Fig. 3).

### Proposed diet for designing SRD based on body-size distribution and survivability

**Figure 4:**
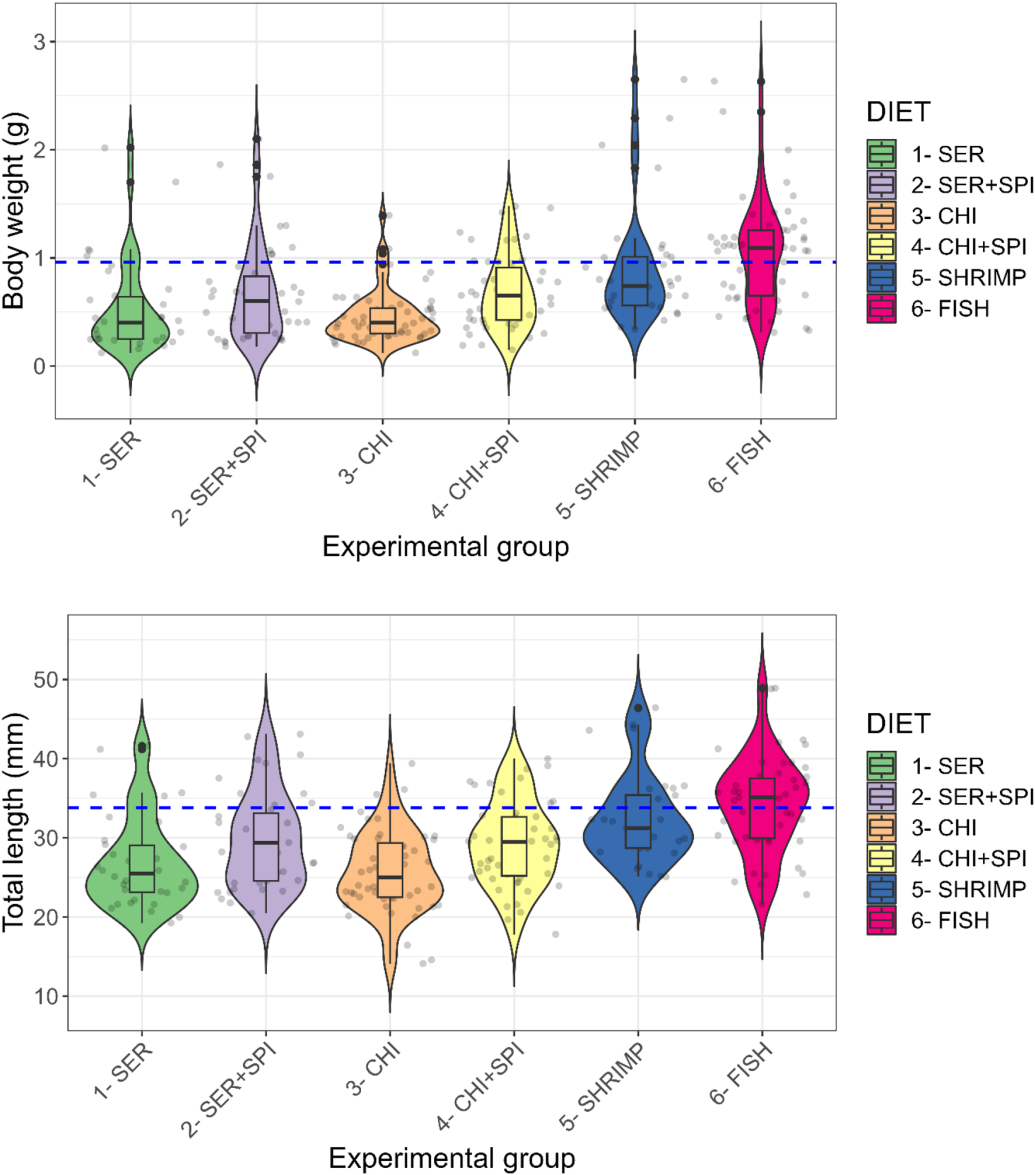
Final body weight, total length and their distribution after 100 days ad libitum feeding of experimental diets at 21 °C (initial body weight 0.03 g). Transparent jitters are individuals. Blue horizontal line is the benchmark for the top 25% of individuals in terms of body weight (≥ 0.96 g) or total length (≥ 33.8 mm). FISH diet was significantly different (*p* < *0.05*) from the other diets.

Diet and replicate-wise measured final body weights, final carapace and total lengths, as well as data at stocking, are provided in Supplementary ﻿information Table S6. Body weight ≥ 0.96 g and total length ≥ 33.8 mm represented the thresholds for the top 25% largest individuals in all experimental groups after 100 days ad libitum feeding at 21 °C. The top 25% individuals were mostly in the FISH diet (34.07 ± 5.96 mm total length; 1.04 ± 0.48 g body weight) (Fig. 4). Compared to FISH diet, the mean body weights using traditional lab feeds (SER or CHI; Tukey HSD *p* > *0.05*) were, respectively, ~ 3 times lower (Tukey HSD *p* < *0.05*) or ~ 1.5 times lower in total length (Tukey HSD *p* < *0.05*).

Whenever the traditional diet was fortified with spirulina (to enhance the leucine supply)—as per the ideal protein concept of wild marbled crayfish (see Supplementary ﻿information Table S4)—there was a statistically significant increase in total length (but not body weight). For example, the total length in CHI + SPI and SER + SPI was significantly higher than CHI (Dunn-Bonferroni test *p* < *0.01*) or SER (Dunn-Bonferroni test *p* < *0.05*), respectively. This implies that compliance with the ideal protein concept was significant. Overall, the FISH diet had a significantly higher (Dunn-Bonferroni test *p* < *0.01*) total length of individuals than all the other diets (Fig. 4).

In terms of size and weight distribution, the violin plot (Fig. 4) depicts a population skewed in the SER, CHI and SHRIMP groups. The most homogenously distributed population was in CHI + SPI followed by the FISH and SER + SPI groups (Fig. 4). Survivability exceeded > 80% in the FISH diet. The lowest survivability (< 60%) was observed in SER and SHRIMP (Supplementary ﻿information Fig. S2). Sexual maturity (presence of glair gland) and gravid females with eggs appeared most often in FISH followed by the SHRIMP diet (Supplementary ﻿information Table S6).

The overall results suggest that following the FISH diet is the optimal strategy for marbled crayfish SRD protein, although the estimated average requirement (EAR) of protein in marbled crayfish SRD could be between SER + SPI (41% protein) and CHI + SPI (47% protein) diets. These two diets had the second highest frequencies (n = 9, 10) for the top 25% individuals and homogenous size distribution after the FISH diet (Fig. 4). The EAR for marbled crayfish SRD protein is therefore recommended to be ~ 44% to give the nearest possible performance to the FISH diet (which could be seen as the upper limit).

## Discussion

The development of SRD for laboratory model organisms is a time-consuming and multi-step process. It involves several rounds of manipulative experiments, employing one nutrient or one energy source at a time, and the control of other variables using easily bioavailable semi-purified research ingredients with standardized compositions ^3^. This process can take up to a decade or two as in the zebrafish model^2^. However, the marbled crayfish has not yet been the subject of such nutritional studies. Therefore, their optimum feed composition is largely unknown. On the other hand, some preliminary insights have already been noted for preferred feed composition when a wide range of practical diets are tested^12^. The present findings could provide baselines for future SRD development in tandem with the nutritional findings for other extensively studied crustaceans (see below).

Our analysis hints at a preferably ketogenic diet preference for marbled crayfish, characterized by a high protein (including phosphorus) intake and energy derived from protein and lipids but not starch (carbohydrates). Indeed, in the kuruma prawn *Penaeus japonicus*, proteins are the main energy source, as observed during starvation ^26^. When feeding, it has been hypothesized that proteins (followed by lipids) are generally superior to carbohydrates as an energy source in the giant river prawn *Macrobrachium rosenbergii* ^27^. The crustaceans tend to be lean^28^. In shrimps and prawns, an excess of dietary non-protein (energy) fractions such as lipids and carbohydrates has been shown to inhibit growth or survival, as reviewed by D`Abramo^29^. There is a preponderance of protein essentiality rather than non-protein energy essentiality in procambarid crayfish^30,31^. Previous research^31^ used menhaden fish oil as a lipid source (0–15% in 3% increments) for white river crayfish *Procambarus acutus acutus* and found less growth in crayfish fed on diets containing ≥ 9% lipids. The negative responses of crustaceans to plant-based diets has been attributed to the presence of phytochemicals including anti-nutritional factors, phytoestrogens, dietary fibres, enzyme inhibitors and a “high carbohydrate content”^27^. Therefore, the FISH diet or CHI + SPI diets (high in protein, low in carbohydrates) could represent a good model for dietary macronutrients and energy composition for marbled crayfish SRD.

The protein fraction is the most important fraction for marbled crayfish SRD. Preliminary efforts in terms of dietary protein optimization in marbled crayfish have focused traditionally on supplementing with more methionine^22^. For fish, lysine (Lys) and methionine (Met) are usually the limiting IAAs that can retard growth^8^. Arginine (Arg) is perhaps the most limiting IAA in crustaceans such as shrimps^32,33^ and red swamp crayfish *Procambarus clarkii*^21^. Arg is involved in protein synthesis, endogenous growth-hormone secretion (or the ecdysone hormone in crustacea; analogous to vertebrate growth hormones), enhanced energy production and body fluid circulation^34,35^. Thus, there is a certain degree of divergence within aquatic animals between vertebrate models (fish) and invertebrate models (crustaceans). In the present study, diets that had Arg > Lys (FISH) or Arg ≈ Lys (CHI + SPI, SER + SPI) were explored as ideal template(s) for SRD or EAR development for marbled crayfish, respectively. Additionally, a uniquely high preponderance of leucine (Leu) in wild marbled crayfish whole-body amino acid composition (that also had lysine ≈ arginine)^36^ could also mean that Leu is a critical IAA in marbled crayfish dietary protein. Previously, although it had only been reported in short sarcomere fibres in the Australian common yabby *Cherax destructor*, leucine may comprise up to 11–12% of total amino acids and arginine 20–21%^37^. Together, they may comprise up to one third of the total amino acid pool in sarcomeres. Leucine is a central amino acid forming the backbone of glycoprotein chitin, and crustaceans assimilate it efficiently when they synthesize cuticular proteins^38–40^. Based on our results, we believe that Leu and Arg, followed by Lys and Met are the highest priority IAAs when designing SRD protein for marbled crayfish. A standard methodology using semi-purified research diets (with crystalline amino acids supplementation supplied one at a time) for standardizing the Arg, Leu, Lys and Met requirements of marbled crayfish is still needed to validate our proposed optimum requirement (i.e. arginine 3%, leucine 4%, lysine 3.5% and methionine 1.2%).

The present study shows that there could be benefits when designing proteins in SRDs for marbled crayfish due to the ideal protein concept of the species^41^. Our dietary evaluation (a) confirmed previous findings regarding arginine essentiality in a procambarid crayfish^21^; (b) complied with the high leucine make-up in natural whole-body protein (*vis-à-vis* presumably high metabolic demand)^36^; and (c) confirmed lysine-methionine as a limiting IAA in aquatic species^42^. The FISH diet protein resembled most closely the wild marbled crayfish whole-body amino acid composition^36^, which had lysine ≈ arginine and a higher level of leucine. However, an excess of arginine (as in the SHRIMP diet) could create more dominant and outlier individuals (Fig. 3), with aggressive behaviour (cannibalism) and low survivability (60%). Excess dietary arginine may result in high biological storage of energy such as phosphoarginine in haemolymph^43^, leading to arginine-mediated aggressive behaviour^44,45^. Sulphur-mediated toxicity may also arise due to excess dietary methionine ^46^ (maximum in SHRIMP diet). As well, the FISH diet contained 56% protein, which is quite high to be proposed as a recommended level. We proposed an estimated average protein requirement falling in between CHI + SPI and SER + SPI would be more economic. However, the use of casein-gelatine-based iso-calorific, iso-composition semi-purified research diets with graded protein increments (at the expense of carboxymethyl cellulose) may further validate this.

Designing feed using the ideal protein concept alone has recently been criticised^47^. The ideal protein concept usually considers designing feed by focusing on supplying in the right proportions and quantity only the IAAs that animals are unable to synthesize de novo. However, studies over the past three decades have shown that sufficient dietary dispensable amino acids (which animals can synthesize de novo) such as glutamine, glutamate, glycine and proline are necessary for maximum growth and optimum health in pigs, chickens and fish^47^. The SRD_protein_ for marbled crayfish also represents a conglomerate of 18–20 amino acids that contains the required dispensable amino acids such as the glutamic acid and aspartic acid in quite high amounts, as is evident from the reserves shown in the body composition data (Table 2; Glu + Asp together comprise 28.5% of body protein). To confirm the suitability of a SRD protein for marbled crayfish on a day-to-day basis, these amino acids may need to be more fully explored. For example, the FISH diet provided the highest glutamic acid but not aspartic acid.

**Table 2:** Amino acids composition (except tryptophan) of the experimental diets used in the present study.

Amino acids (g 1000 g^−1^ feed)	SER feed	SER + SPI feed	CHI feed	CHI + SPI feed	SHRIMP feed	FISH feed	Crayfish body
Alanine (ALA)	19.84	23.37	33.64	36.54	35.05	33.84	22.05
Arginine (ARG)*	19.56	22.72	23.08	27.19	34.18	32.16	18.37
Aspartic acid (ASP)	28.49	31.60	53.35	51.33	52.29	49.32	63.28
Cysteine (CYS)	4.70	4.84	3.78	3.81	5.21	5.65	4.90
Glutamic acid (GLU)	102.04	102.48	76.03	74.37	101.75	119.78	58.55
Glycine (GLY)	19.08	20.36	18.98	21.76	39.15	34.46	22.88
Histidine (HIS)*	9.45	9.63	16.24	13.93	15.21	14.61	9.22
Isoleucine (ILE)*	16.13	18.16	20.38	23.52	23.96	23.88	21.78
Leucine (LEU)*	29.39	32.24	30.82	35.46	42.62	43.81	76.05
Lysine (LYS)*	19.96	21.24	25.63	24.50	39.90	36.80	27.68
Methionine (MET)*	7.24	7.95	6.16	7.20	14.09	11.96	10.88
Phenylalanine (PHE)*	17.84	18.87	25.61	23.47	23.32	24.06	15.38
Proline (PRO)	30.34	28.72	53.50	38.68	32.32	35.25	5.72
Serine (SER)	19.80	19.90	21.32	22.82	26.17	25.72	15.83
Threonine (THR)*	13.90	16.52	19.42	21.32	24.12	23.41	17.23
Tyrosine (TYR)	12.04	14.34	13.80	16.79	17.82	17.88	13.17
Valine (VAL)*	18.62	20.94	21.83	25.85	27.70	27.52	21.42
Total amino acids	388.42	413.89	463.60	468.53	554.87	560.09	426.32

All values are expressed on a 100% dry-matter basis. All feeds had consistent dry-matter values (91–93%). Wild-caught marbled crayfish *Procambarus virginalis* whole-body homogenate dry-matter amino acids are given as ‘Crayfish’ reference. The highest indispensable amino acid is highlighted in grey.

*Indispensable amino acids (IAA). SER = Sera Granugreen; SER + SPI = Sera Granugreen + Spirulina; CHI = Chironomids; CHI + SPI = Chironomids + Spirulina; FISH = Fish feed; SHRIMP = Shrimp feed.

The present study provides the foundation for marbled crayfish SRD development and aims to fast track its progress. Some limitations of the study are also discussed. Traditionally, for laboratory models, feed is usually provided ad libitum, whereby animals have unrestricted access to food and its availability is not constrained. The present study used such an approach, which did not allow for the computation of the feed conversion ratio (FCR) or the protein efficiency of the tested diet(s). It may be noted that the protein efficiency may be higher in constrained feed rationing. Another limitation of this study was that anthropometric measurements indicate overall growth but not the metabolic status, for which metabolomic- or bioenergetics-based studies would be necessary (also under constrained feeding). It may be difficult to employ the latter approach with crayfish kept in community tanks since cannibalism could add to the nutrient/energy intake (apart from feed provided). In addition, animal species have different nutritional requirements with different life stages. Our experiment started with very small individuals with a presumably high nutritional (protein, amino acids) requirement that gradually decreased with increasing body size. Although our animals grew 32 times from their initial body weight in 100 days, we only tested a single diet so as not to introduce another variable (feed) into the study. Indeed, life-stage-specific SRDs are used as in zebrafish (e.g. GEMMA feed line). Simultaneously the varied macronutrient composition may affect the interpretation of the findings, especially in terms of requirements. However, the relationship trends presented here via the exploratory data analysis and corroborated through literature on crustacean nutrition do provide baselines for future SRD development. This will require additional rounds of experiments and validations of semi-purified research diets, as proposed above.

## Methods

### Design and preparation of experimental feed

Step 1. Three diets traditionally used in laboratories (two ornamental fish feed: Sera Granugreen, Tetra wafer mix; one natural food type: frozen chironomid larvae) were assayed on experimental specimens for their amino acids profile (Supplementary ﻿information Table S1) and on wild-caught individuals from the Czech Republic, Slovakia and Hungary (Supplementary ﻿information Table S2). The amino acids analyses were performed in an accredited third-party laboratory (Laboratoř Postoloprty s.r.o., Czech Republic) using certified methodologies (Commission Regulation EC No. 152/2009, Annex III, procedure F), as described in a previous study^36^. A total of 18 amino acids (AAs) were quantified: methionine (Met), lysine (Lys), threonine (Thr), aspartic acid (Asp), serine (Ser), glutamic acid (Glu), glycine (Gly), alanine (Ala), tyrosine (Tyr), valine (Val), phenylalanine (Phe), isoleucine (Ile), leucine (Leu), histidine (His), arginine (Arg), cysteine (Cys), proline (Pro) and tryptophan (Try). The results for all the amino acids were accredited with ± 15% uncertainty, except for tryptophan whose result was non-accredited. The quantity of nine indispensable or essential amino acids (Arg, His, Leu, Ile, Lys, Met, Phe, Thr and Val) was converted to % of Lys to explore the ‘ideal protein concept’ for marbled crayfish. The ideal protein concept (based on wild body amino acids profile) was cross-matched with that of traditionally used diets amino acids profile (converted to % of lysine). This suggests that the traditional diets used in laboratories lack leucine supply and may be insufficiently balanced in arginine, a critical AA for procambarid crayfish^21^. Detailed calculations can be found in the Supplementary data Table S3.

Step 2. From the International Aquaculture Feed Formulation Database (IAFFD, https://www.iaffd.com/), naturally occurring protein sources (likely to be encountered in crayfish habitat) were scoped that had a high content of both leucine (around 10% Leu of total amino acids, TAA) and arginine (~ 7% Arg of TAA). The protein source identified was a filamentous algae, *Arthrospira platensis* (spirulina). Hence, dried spirulina powder with the given specification (protein content > 60%) was procured from Trouw Nutrition Biofaktory s.r.o., Prague. Data on the detailed composition of all ingredients used in the preparation of the experimental diets can be found in the Supplementary data (Tables S8–S13).

Step 3. One of either the ornamental fish feed (Sera Ganugreen) and live food (chironomid larvae), both with known amino acid profiles, were chosen for fortification with spirulina. For this purpose, a licensed feed formulation software was used (WinFeed 2.8). The fortification was planned in a near iso-protein, near iso-energy-to-protein ratio manner targeting a relative increase in the Leu + Arg share in TAA, while randomizing the changes in other amino acids relative to the TAA pool. As spirulina is deficient in Calcium (Ca), CaCO_3_ was added to the fortified diets. The composition of each diet is given in Supplementary data (Tables S8–S13). The calorific value of proteins, lipids and carbohydrates was taken as 5.64 kcal, 9.44 kcal and 4.11 kcal per g, respectively^48^.

Step 4. Three protein levels were formed with amino acid manipulations nested in each level (i.e. diets with near similar protein levels but differing in amino acid compositions) to enable the statistical evaluation of which amino acids show the highest power per unit degree of change in the somatic growth response. At the first level (~ 40% crude protein; ~ 11.9 kcal gross energy g^−1^ crude protein), Sera granugreen (SER) and Sera granugreen was fortified with spirulina (SER + SPI). The Leu + Arg shares in SER and SER + SPI were 10.5% and 13.8% of TAA, respectively. At the second level (~ 46% crude protein; ~ 8.7 kcal gross energy g^−1^ crude protein), the lyophilized chironomid larvae (CHI) and chironomid larvae were fortified with spirulina (CHI + SPI). The Leu + Arg shares in CHI and CHI + SPI were 11.6% and 13.4% of TAA, respectively. At the third level (~ 56% crude protein; ~ 9.3 kcal gross energy g^−1^ crude protein), there were two commercially standardized larval diets, one for carnivorous fish (Skretting Perla larva 0.3–0.5 mm; thereafter FISH) and the other for postlarval shrimps (Skretting Shrimp feed PL #3, 300–550 mm; hereafter SHRIMP). The Leu + Arg shares in FISH and SHRIMP were 13.6% and 13.8% of TAA, respectively, similar to the fortified diets (SER + SPI or CHI + SPI) but with Lys and Met in higher proportions.

Step 5. All ingredients (SER, CHI, spirulina) were carefully weighed according to the recipes (see Supplementary data Table S4); the commercial larval diets (FISH, SHRIMP) were directly re-pelletized. In total, 500 g of each diet was targeted for production. Only the chironomid needed pre-processing by lyophilization (18 h main drying + 14 h final drying in a vacuum and -60 °C cooling temperature per 500 g wet mass) to convert wet matter to dry matter. All dry matrices were mixed in a mixer for 30 s, then slowly dosed with 125 ml water for another minute to make a moist dough. A single-screw cold pelletizer (noodle-maker) was used, fitted with a motorized rotating knife cutter to produce pellets that were ~ 5 mm long and 1.5–2 mm in diameter (lowest limit of the machine). Pellets were dried at room temperature (with a fan) overnight and then for 48 h in a hot air oven at 45 °C. Then, they were cooled to room temperature and refrigerated at 4–6 °C until future use. Before being applied to the crayfish tanks, pellets were mechanically crushed to form small grits (i.e. not entirely powdered). All pelleted diets were analyzed for proximate composition, amino acids, calcium and phosphorus in a third-party accredited laboratory (AGROLA, spol. s.r.o., Czech Republic https://agrola.cz/laborator/) using ISO/EU certified protocols. The methods include dry matter (method: ČSN ISO 11,465), ash (ČSN ISO 11,465), phosphorus (ČSN EN ISO 11,885), lipid (ČSN 46 7092–7), fibre (ČSN ISO 6541), protein (ČSN EN 16,634–1) and calcium (ČSN EN ISO 11885). The nitrogen-free extract (NFE) was calculated as NFE = dry matter—(protein + lipid + fiber + ash). Amino acids were analyzed as mentioned above.

The macronutrient composition of the experimental diets is given in Table 1, while the amino acid composition is given in Table 2.

### Crayfish keeping and husbandry

In total, 360 juvenile marbled crayfish with a mean weight of 0.03 ± 0.01 g were used as experimental animals (20 individuals per aquaria; six groups in triplicates). The experiment was performed for up to 100 days in a series of indoor glass aquaria (54 × 36 × 30 cm, volume 46 L) in a system with water recirculation. Altogether, 18 aquariums were subjected to stable climatic conditions (21 ± 1 °C) with an artificially maintained photo regime (12L:12D). One brick (28.5 × 13.5 × 6.5 cm) with 39 cross holes (26 and 13 holes with a profile of 1 × 3 cm and 1 × 1 cm, respectively) was placed in each aquarium to provide shelter. Additionally, polypropylene pipes containing five tubes (length 10 cm, inner diameter 35 mm) were placed in each aquarium as additional shelter for still-growing animals^21^.

Marbled crayfish juveniles were fed for one hundred days with the abovementioned diets ad libitum twice per day to apparent satiation (6% of the body weight; at 08:00 h and 15:00 h). Feed was usually spread over a wide-open space in the tank, rather than heaped in one place to ensure equal access by all animals and to preclude feed-induced aggression. Uneaten feed, feces and other waste matter was siphoned out manually each morning. Dissolved oxygen (8.9 ± 0.5 mg L^−1^), pH (7.3 ± 0.3) and temperature (21 ± 1 °C) were measured daily using Oxi 3205 and pH 720 m (WTW GmbH, Weilheim, Germany), respectively. The body weight of marbled crayfish from each aquarium was measured every 14 days using an electronic balance (lowest sensitivity 1 mg) and the number of survivors was counted. Body weight measurements were taken before feeding. At the end of the experiment, the animals were starved for one day and their final body weight was taken. Total body and carapace lengths were also recorded (Supplementary data Table S6).

No specific authorizations were required for the location and activities involved in this study. Based on the EU harmonized animal welfare act of Czech Republic, all manipulations with organisms were approved by the Institutional Animal Care and Use Committee (IACUC) of the University of South Bohemia, Faculty of Fisheries and Protection of Waters, Research Institute of Fish Culture and Hydrobiology, Vodňany. The principles of laboratory animal care and the national laws 246/1992 and regulations on animal welfare were followed (Ref. number 22761/2009-17210).

### Statistical analysis and interpretation framework

Animals were stocked randomly and a parametric test (one-way ANOVA) confirmed that there was no significant difference in body weight between groups at the beginning of the experiment. Intermittent measurements were taken at fortnightly intervals. At the final time point (100 days), the number of individuals headcount and their body weights per tank were taken as the final observation. Following this, three response variables were calculated: specific growth rate (SGR)^49^, final body weight, and survivability^21^. Additionally, a visual inspection of the appearance of the glair gland and egg attachment was conducted. The corrected formula for SGR was used^49^: SGR = 100*(e^g^ − 1), where ∆t = 100 days and e^g^ = (w_2_ ÷ w_1_)^1/∆t^. Here, w_2_ = median final body weight (in grams, g) and w_1_ = median initial body weight (in grams, g). To avoid bias emanating from a social hierarchy-based size distribution specific to each tank, a median was used as a measure of central tendency rather than the mean value^21^. As such, SGR was calculated replicate-wise (in triplicate). Statistically significant differences in SGR between protein levels or diets were determined at a set-alpha level 0.05. The grouped data were first evaluated using Shapiro–Wilk’s normality test; following the *p-value*, one-way ANOVA with *post-hoc* Tukey HSD (parametric test) or Kruskal–Wallis *post-hoc* Dunn’s test with Bonferroni correction (non-parametric test) was selected. A multi-variate analysis to identify the most significant nutrient or energy parameter (independent variables) influencing SGR was performed first using a multiple linear regression (MLR) in an ANCOVA framework^50^, followed by a refinement of the model using stepwise regression (both forward and backward combined; *i.e.,* bi-directional).

Given that the influence of the protein on SGR was identified (fulfilled), further statistical evaluation of the group of 18 dietary amino acids (making up the dietary protein) was performed. Here, in addition to SGR (which identifies the rate of somatic investment), the final individual body weight was taken as an additional response variable (to measure the absolute amount of somatic investment from dietary amino acids to body protein). Furthermore, a principal component analysis (PCA) biplot identifying multicollinearity between amino acids and the SGR, as well as bivariate generalized linear models (GLM) of dietary indispensable amino acids (IAA; predictor variable) and the body weight of marbled crayfish (response variable), were evaluated. Individually, the goodness-of-fit of the GLM(s) was determined by McFadden’s R^2^ and *p*-value by a default summary function-fitted GLM model in R. The *p*-value was evaluated more strictly than the macronutrients at an alpha level established at 0.01. The *ggplot2* package was used to visually present the results. The GLM model was interpreted using a set benchmark for the top 25% body weight encountered in the experiment, that is, the likelihood/frequency of an individual above the 75^th^ percentile body weight of the whole experimental stock appearing in a particular diet(s). Survivability was tracked for the duration of the experiment and across diets using a Kaplan–Meier survival analysis in *ggplot2*. All analysis was done in RStudio Double Marigold. Additionally, it was determined whether a statistically significant increase in body weight (for a particular diet) was concomitant with an increase in total length to discriminate overfed and heavy specimens from evenly growing individuals.

### Supplementary Information

Supplementary Information.

## Data Availability

All the data used in this manuscript and supplementary data are made freely available.
